# Hedgehog pathway, cell cycle, and primary cilium

**DOI:** 10.1038/s41420-025-02605-7

**Published:** 2025-07-03

**Authors:** Tenghan Zhuang

**Affiliations:** 1 Institute of Veterinary Immunology and Engineering, Jiangsu Academy of Agricultural Sciences, Nanjing, Jiangsu China; 2 GuoTai (Taizhou) Center of Technology Innovation for Veterinary Biologicals, Taizhou, Jiangsu China

**Keywords:** Cell signalling, Cell-cycle exit, Organelles

## Abstract

The Hedgehog (Hh) pathway was initially identified as essential for development and tumorigenesis. In addition to its well-established and indispensable roles within the primary cilium, some components of this pathway have demonstrated more general functions in cell cycle progression. Therefore, this review aims to summarize recent advancements regarding the crosstalk among the Hh pathway, the primary cilium, and the cell cycle, while also highlighting potential issues that may arise in future research.

## Facts

- Hh pathway participates in embryogenesis, regeneration, homeostasis, and tumorigenesis.
- Hh pathway components and cell cycle members mutually regulate the translocation and function of each other.
- The primary cilium is required for responding to the Hh pathway and is assembled or disassembled in coordination with the cell cycle.

## Open questions

- What is the molecular basis of Sufu repression by Smo, and the regulatory mechanisms governing the sub-ciliary localization of Sufu in response to Smo activity?
- What is the functional difference of the same protein between ciliary localization and outside-of-cilia localization?
- What is the regulatory network of ciliary vesicle release and the selection of the components?
- Cholesterol plays a crucial role in regulating Hh ligand modification, inducing Ptch1 regulation of Smo, and affecting the plasma membrane. Given that cholesterol is widely present in the body, particularly in brain and nerve tissue, as well as in the kidney, spleen, skin, liver, and bile. Whether the widespread distribution of both primary cilia and cholesterol represents a new regulatory network for cell proliferation or signaling transduction?
- Whether cell cycle-related proteins such as CDK4, CDK2, AurA, Plk1, and CDK1 form a regulatory network that determines the fate of the primary cilium?

## Introduction

The *hedgehog (hh)* gene was first identified through a screen of embryonic lethal mutants in *Drosophila* [1], deriving its name from the resemblance of its hair-like bristles to hedgehog spines observed in *hh* null alleles. The Hh family proteins are evolutionarily conserved morphogens that regulate cell positioning and fate during early embryonic development. The mammalian Hedgehog (Hh) family comprises sonic Hh (Shh), Indian Hh (Ihh), and desert Hh (Dhh). Shh is integral to the organization of the central nervous system and the development of limb polarity, while Ihh is crucial for bone and cartilage development. is involved in developing germ cells and forming the peripheral nerve sheath [2–5]. Following its role in embryogenesis, the Hh pathway orchestrates reparative and regenerative responses [6], maintaining stem cell homeostasis in adult tissues [7], including the pancreas [8], liver [9, 10], lungs [11], and skeleton [12].

Misactivation of the Hh pathway is associated with various developmental abnormalities and malignancies, including medulloblastoma (MB), basal cell carcinoma (BCC), rhabdomyosarcoma, nevoid basal cell carcinoma syndrome (Gorlin syndrome), pancreatic ductal adenocarcinoma, lung cancer, prostate cancer, and atypical teratoid rhabdoid tumor [13]. Additionally, mutations or abnormal expression of Hh pathway components are linked to severe conditions such as holoprosencephaly, Greig cephalopolysyndactyly syndrome, hereditary multiple exostoses, polydactyly, encephalocele, neural tube defects, and cerebellar vermis hypoplasia [14].

Consequently, the Hh pathway is a focal point of extensive research in developmental, reparative, regenerative, and cancer biology, as even minor defects within this pathway can lead to a spectrum of diseases, from birth defects to tumorigenesis. This review refers to the Shh as the Hh ligand for overarching concepts. Our objective is to provide recent updates on the Hh pathway, including the crosstalk between the Hh pathway and the cell cycle regulation. We illustrate the unexpected complexity of the molecular mechanisms underlying Hh signaling transduction, particularly how the primary cilium facilitates compartmentalization. Furthermore, we highlight the alterations in the Hh pathway at various stages of the cell cycle and outline its regulatory role in this process. Additionally, we review the assembly and disassembly of the primary cilium in relation to the cell cycle.

## The Hedgehog signaling cascade

Hh proteins are synthesized by epithelial cells as precursors, which are activated through cleavage of the intein domain [15]. Upon binding to the 12-pass transmembrane receptor Patched homolog 1 (Ptch1) [16, 17], the first Hh receptor identified in *Drosophila* [18, 19] that constitutively represses the Hh pathway, Hh proteins initiate the downstream signaling cascade by releasing another 7-pass transmembrane protein, Smoothened (Smo) [20], a member of the class Frizzled G protein-coupled receptor (class F GPCR) superfamily. Subsequently, Smo translocates to the plasma membrane in *Drosophila* [21, 22] and to the primary cilium in vertebrates [23] to activate the downstream pathway [24]. Activated Smo then engages the only known transcriptional mediators of the Hh response, the Gli family [Cubitus interruptus (Ci) in *Drosophila*] of zinc-finger proteins, including the primary activator Gli2 [25], the main repressor Gli3 [26], and a feed-forward transcriptional activation amplifier Gli1 [27]. In the absence of the Hh ligand, the Suppressor of fused (Sufu) binds directly to the Gli proteins [28–33], regulating their nuclear localization [33–36].

### Biosynthesis, transport, recognition, and inhibition on Patched of the Hedgehog ligand

The production and secretion of active Hh proteins entail several processes: including autocleavage processing, modification by palmitate and cholesterol, recycling and packaging in the microvesicles, and spreading through cytosolic structures, including cytonemes and extracellular exosomes [14, 37].

All Hh polypeptides are directed into the endoplasmic reticulum and Golgi apparatus, where they undergo cleavage into two fragments [38, 39]: the amino-terminal fragment, which acts as a signaling molecule [40], and the carboxyl-terminal fragment, possessing intein-like activity [41]. The amino-terminal segment of the Hh protein is further modified with palmitate and cholesterol, which enhance its hydrophobicity and stability within the extracellular matrix [42, 43]. The palmitoylation is mediated by O-acyltransferases, such as Skinny Hh (Ski) in *Drosophila* [44] or Hh acyltransferase (HhAT) in vertebrates [45]. In the final stage of the generating extracellular mature Hh proteins, the release of these proteins, which have undergone dual lipid modification [46], is facilitated by Dispatched (Disp) in *Drosophila* and Disp1 in vertebrates [47], along with the vertebrate-specific Scube 2 [48]. The absence of cholesterol or palmitate results in defects in the formation of the Hh protein multimer, thereby compromising long-range signaling and transduction capabilities [42, 49–51]. Additionally, Hh proteins located at the plasma membrane may be recycled and subsequently released via exosomes derived from the multivesicular body (MVB), a process that is supported by endosomal sorting complexes required for transport (ESCRT) [52–55]. Furthermore, cytonemes, which are specialized filopodial structures formed in Hh-producing cells, play a pivotal role in mediating the transport of the Hh signal to distant regions within the tissues [56–59].

Upon reaching the responding cell membrane, Hh proteins engage with Ptch1, which directly interacts with them to form a complex that translocates from the primary cilium to the plasma membrane [16, 17]. Ptch1 inhibits Smo activity by inducing its rapid degradation and/or confining it to an intracellular compartment in *Drosophila* [20], while in vertebrates, it prevents Smo accumulation within the primary cilium [60]. The palmitoylation of Hh protein is essential for Ptch1 inhibition in both *Drosophila* [44] and vertebrates [44, 61]. Notably, a short palmitoylated amino-terminal peptide of Hh has been identified as sufficient for activating the Hh pathway and inhibiting Ptch1 in vertebrates [62]. Although this peptide mimics the inhibitory effects of the full-length Hh protein on Ptch1, it exhibits distinct effects on Ptch1 trafficking, degradation, and endocytosis [63]. This observation is consistent with the notion that the dynamin-dependent turnover of Ptch1 is not requisite for Hh pathway activation [64], indicating that the internalization of Ptch1 alone does not suffice for its inhibition.

In addition to Ptch1, several other co-receptors modulate the Hh pathway, including positive co-receptors such as cell-adhesion molecule-related, downregulated by oncogenes (Cdo), brother of Cdo (Boc), and the vertebrate-specific growth arrest-specific 1 (Gas1). The pathway also involves a negative co-receptor like Hedgehog-interacting protein (Hhip), as well as dual-function co-receptors heparan sulfate proteoglycans (HSPGs) [37, 65]. The presence of multiple co-receptors that regulate responses to Hh proteins allows for the orchestration of both the range and intensity of Hh signaling responses across various tissues. This complex network of interactions underscores the importance of co-receptor diversity in fine-tuning Hh signaling in developmental and physiological contexts.

### Regulation of Smoothened by Patched

In the absence of the Hh ligand, Ptch1 is localized at the plasma membrane in *Drosophila* or concentrated within the primary cilium in vertebrates, while Smo undergoes degradation in *Drosophila* or is sequestered in an endomembrane compartment away from the primary cilium in vertebrates. Upon stimulation by Hh, Ptch1 is endocytosed and subsequently degraded, resulting in the accumulation of Smo at the plasma membrane in *Drosophila* or within the primary cilium in vertebrates. The biochemical mechanisms underlying the inhibitory effects of Ptch1 on Smo have remained inadequately understood for nearly 25 years. Following Hh ligand binding and Ptch1 degradation, Smo becomes phosphorylated by Casein kinase 1 (CK1), G-protein-coupled receptor kinase 2 (GRK2), transitioning into an activated conformation within the primary cilium in vertebrates [66]. Research by Chen and Jiang suggests that varying levels of Smo phosphorylation may facilitate the interpretation of Hh gradients through a phosphorylation code in *Drosophila* [67].

In the initial model, Ptch1 was thought to inhibit Smo through direct binding, with Hh ligand-induced disassociation of the Ptch1-Smo complex leading to Smo activation [17, 68]. However, this model was reconsidered following the discovery of distinct subcellular distributions of Ptch1 and Smo in both *Drosophila* and vertebrates [20, 23, 60]. Given the structural similarities between Ptch1 and bacterial resistance-nodulation-division (RND) permeases, which expel toxic substances from cells using proton gradients [69, 70], and catalytically suppression of Ptch1 on Smo in both *Drosophila* [71] and vertebrates [72], a new ion-driven pump model emerged. In this model, Ptch1 exerts its inhibitory effect on Smo by pumping out ion-like Smo agonists. This hypothesis is compelling due to its alignment with the observation that Smo is regulated by endogenous metabolites, including vitamin-D3 [73, 74].

**Fig. 1:**
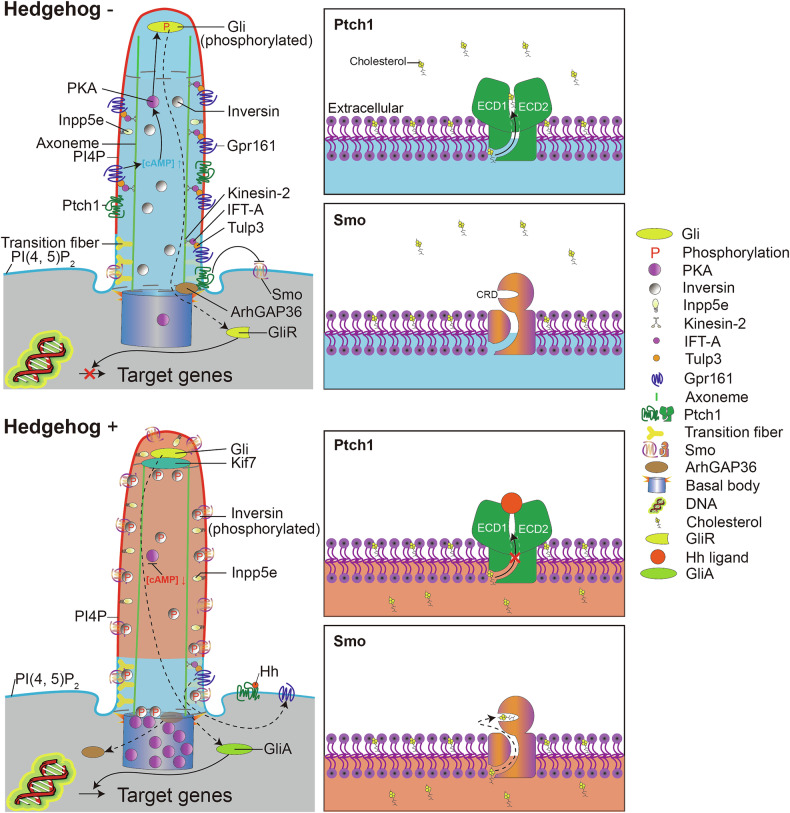
Overview of vertebrate Hedgehog pathway. Hedgehog (Hh) pathway patterns limbs and the neural tube. As the most studied ciliary signaling pathway, the Hh pathway interacts with the ciliary environment to trigger a transcriptional response. In Hh unstimulated cells (upper left panel), the main receptor of the Hh pathway-Patched1 (Ptch1, green) binds to and stabilizes the PKA negative regulator ArhGAP36 (brown) to the centrosomal area, which results in a low-level of centrosomal PKA (gradual magenta). Ciliary Ptch1 inhibits ciliary localization of the main transducer of the Hh pathway-Smo (violet gold), which induces the ciliary accumulation of G-protein-coupled receptor 161 (Gpr161, Ming blue) with the help of intraflagellar transport (IFT)-A (shadow magenta), Kinesin-2 (light brown), and Tubby-like protein 3 (Tulp3, orange). Ciliary Gpr161 then leads to elevated cyclic adenosine monophosphate (cAMP) concentration and activation of ciliary PKA. Phosphorylation (P) of the transcription effectors Gli (Kelly yellow) proteins by PKA inside the primary cilium puts them to proteolytic processing into transcription repressors (GliR, Kelly yellow), which induces low expression of target genes. Upon Hh stimulation (bottom left panel), binding of Hh ligand (bright orange) to Ptch1 results in Ptch1 removal from the primary cilium, the release of Ptch1 inhibition on Smo, and elimination of centrosomal ArhGAP36, which liberates centrosomal accumulation of PKA. Accumulated centrosomal PKA then phosphorylates inversin (gradient black and white). Phosphorylated (P) inversin interacts with Smo and promotes its ciliary translocation and expands the inversin compartment (gray) to a more distal area compared with the Hh-unstimulated situation. Meanwhile, inositol phosphoinositide 5-phosphatase e (Inpp5e) dephosphorylates ciliary PI(4, 5)P_2_ into PI(4)P and thus establishes a PI(4)P compartment (blue), where ciliary Smo determines β-arrestin-mediated removal of the ciliary Gpr161 that facilitates downregulation of ciliary cAMP concentration and inactivation of ciliary PKA. Hence, without phosphorylation by PKA, Gli translocates into the nucleus in a transcription activator form (GliA, Kelly green) without undergoing proteolytic processing to promote downstream target gene expression. In the absence of Hh (upper right 2 panels), Ptch1 transports cholesterol out of the cytoplasmic leaflet with its hydrophobic tunnel that courses from the cytoplasmic leaflet to the extracellular domain (ECD), which lowers the concentration of cholesterol in the primary cilium. In parallel, there is also a hydrophobic tunnel of Smo that may allow cholesterol to be transported from the inner leaflet to its cysteine-rich domain (CRD), and cholesterol binding to the CRD is sufficient to activate Smo. Thus, low cholesterol levels inside the ciliary membrane inhibit Smo activation. The tunnel of Ptch1 becomes obstructed upon binding of the Hh ligand to Ptch1 (bottom right 2 panels), which leads to increased cholesterol concentration in the primary cilium. Constant trafficking of Smo in and out of the primary cilium guarantees that the elevated cholesterol level is rapidly sensed by Smo and that the binding of cholesterol to CRD of Smo activates downstream signaling transduction.

Further insights into the Hh pathway arise from investigations in vertebrates. Despite its role in mediating modification at the carboxyl-terminal of Hh proteins [75], cholesterol itself, rather than any precursor or product sterol, was identified as essential for the Hh pathway through a targeted loss-of-function screen encompassing all lipid-related genes, including those involved in sterol and steroid synthesis [76]. Structural studies reveal a sequence similarity between Ptch1 and the cholesterol transporter Niemann–Pick C1 (NPC1) as well [62, 74, 77–80]. Both proteins possess a sterol-sensing domain (SSD) that is crucial for cholesterol handling or sensing [81–85]. Additionally, cholesterol activates Smo by binding to a site within its cysteine-rich domain (CRD), a binding disrupted by CRD mutations [86–88]. Consequently, the model of Ptch1 inhibition of Smo has evolved to propose that cholesterol functions as a second messenger mediating communication between Ptch1 and Smo. A predicted tunnel within Ptch1 may facilitate the transport of cholesterol from the inner to the outer leaflet of the cell membrane, leading to reduced cholesterol concentration and subsequent inactivation of Smo [78–80] (Fig. 1).

In our recent study, we identified Ptch1-ArhGAP36-protein kinase A (PKA)-inversin-Smo axis that provides a refined understanding of how Ptch1 regulates the translocation of Smo into the primary cilium [89]. Ptch1 stabilizes ArhGAP36, which inhibits PKA activity and directs the degradation of the PKA catalytic subunit (PKAc) at the mother centriole in the absence of Hh ligand [90]. Thus, the diminished levels and activity of centrosomal PKA are insufficient to efficiently phosphorylate inversin, thereby hindering its ability to bind to Smo and sequester Smo within the inversin compartment of the primary cilium (Fig. 1). This finding challenges the prevailing notion that PKA exerts a negative influence on the Hh pathway, as evidenced by increased Gli expression under pharmacological inhibition or genetical depletion of PKA [67, 91, 92]. However, previous studies demonstrating negative regulation of the Hh pathway by PKA involved universal treatments, suggesting that the spatiotemporal role of PKA within the primary cilium or at the centrosome may indeed be reversed. Ultimately, Ptch1 represses Smo activity via its cholesterol-pumping mechanism and facilitates Smo translocation to the inversin compartment by stabilizing the centrosomal PKA inhibitor ArhGAP36.

While the roles of Smo in regulating Gli transcription factors, calcium flux, and metabolic pathways have been extensively reviewed [93], the downstream signaling relay of the Hh pathway following Smo activation remains incompletely understood. Further investigations are necessary to elucidate the mechanisms by which Smo transduces signaling downwards.

### Regulation of Sufu on Gli

*Sufu* was initially identified for its ability to fully suppress both embryonic and adult phenotypes in *fused* (*fu*) mutants of *Drosophila* [94]. The human *SUFU* is located on chromosome 10q24-q25, a region frequently deleted in glioblastomas, prostate cancer, malignant melanoma, and endometrial cancer, where it functions to repress the activity of the zinc-finger transcription Gli [30]. While depletion of *Sufu* does not lead to lethality in *Drosophila* [94], ablation of the mouse *Sufu* gene results in embryonic lethality around embryonic day 9.5, accompanied by cephalic and neural tube defects and a constitutive upregulation of Gli expression, resembling the effects observed with *Ptch1* depletion [32]. Research indicates that Sufu can expel Gli from the nucleus [29, 34, 35] and acts as a Gli repressor by associating with Gli in the absence of the primary cilium [95, 96]. The mechanism by which Sufu suppresses Gli involves sequestering Gli in the cytoplasm and inhibiting its activation through binding to the N-terminal Sufu-binding site (SIN) and the C-terminal Sufu-binding site (SIC) of Gli, respectively [97].

*Gli* was identified as an amplified gene in human gliomas [98]. The Gli family comprises vertebrate homologs of the *Drosophila* transcription factor Cubitus interruptus (Ci) [99]. The Gli1, Gli2, and Gli3 proteins exhibit high homology [100] but serve specialized functions within the Hh pathway. Gli1 functions exclusively as a transcriptional activator and is regarded as a sensitive readout of the Hh pathway activity [27, 101, 102]. While Gli2 and Gli3 display dual activities [103], Gli3 primarily acts as a transcriptional repressor, designated as GliR [26], whereas Gli2 serves as a major pathway activator, designated as GliA [104–106]. This functional distinction underscores the complexity of the Hh pathway and the diverse roles played by the Gli family in developmental processes and tumorigenesis.

Activation of the Hh pathway facilitates the translocation of the Sufu-Gli complex into the primary cilium, where dissociation occurs, leading to the accumulation of both Sufu and Gli at the distal end of the cilium. This process allows the GliA to translocate to the nucleus, which drives the transcription of downstream target genes [2, 107–109] (Fig. 1). The formation of the Sufu-Gli complex has been shown to stabilize Gli proteins [29, 30, 110]. In the absence of Hh ligand, PKA and Glycogen synthase kinase 3β (GSK3β) phosphorylate and stabilize Sufu [111], while the Gli proteins are phosphorylated by PKA, CK1, and GSK3β, resulting in partial degradation by proteasomes to yield C-terminal truncated GliR, which then enters the nucleus to repress Hh target gene expression [26, 112]. PKA localizes to the base of the primary cilium to prime Sufu phosphorylation within the Sufu-Gli complex [92]. Upon Hh ligand binding, these phosphorylation events are blocked, leading to the accumulation of Sufu and Gli at the ciliary tip [26, 111, 112]. The phosphorylation status of Sufu dictates its interaction with Gli proteins; non-phosphorylated Sufu binds exclusively to full-length Gli3, but not to Gli3R [107, 113] or GliA [108]. The passage of the Sufu-Gli complex through the primary cilium reflects the phosphorylation state of both proteins, causing Sufu to disassociate with Gli in the cases of phosphorylated Sufu/Gli3 and Sufu/GliA, allowing GliR and GliA to enter the nucleus for target gene transcription.

In addition to its interaction with Gli, the multifunctional Sufu has been reported to engage with several nuclear proteins, including Mycbp and p66β [114], which are involved in the regulation of the Hh pathway. Sufu also associated with Sin3-associated polypeptide 18 (SAP18), Galectin3 [115], protein inhibitor of activated signal transduction and activators of transcription 1 (PIAS1), and p300/CBP-co-integrator protein (pCIP) [116], playing a role in the transcriptional regulation of targets within both the Hh and Wnt pathways. Furthermore, Sufu binds to Sox10 [117] to facilitate neural stem cell differentiation. Notably, nuclear Sufu is subject to ubiquitination and subsequent degradation by SCF^Fbxl17^ [118], highlighting its regulated turnover in cellular signaling processes.

## Hh pathway components regulate cell cycle progression

Over the past two decades, extensive research conducted by developmental and cellular biologists has elucidated the key molecular components and essential functions of the Hh pathway. Recent studies have demonstrated that the Hh pathway engages in crosstalk with the phosphoinositide 3-kinase (PI3K) pathway [119], viral infections [120], and mammalian target of rapamycin complex (mTORC)1 pathway [121]. Investigations into the mechanisms underlying the Hh pathway have revealed novel regulatory aspects pertinent to cell biology. One of the primary functions of the Hh pathway in development is the regulation of cellular proliferation, which serves as a critical driver for cell cycle control [122]. Activation of the Hh pathway leads to the upregulation of transcriptional levels of various cell cycle regulators, including *CCND1/2* [123–126], *CCNE* [124, 125, 127], *Cdk6* [128], *Cdkn1c* [129], *N-Myc* [127], *E2F1* [126, 130], *E2F2* [126], *CDC25* [127]. These findings underscore the pivotal role of the Hh pathway in coordinating cell growth and division.

### Ptch1 interacts with CyclinB1 and regulates its translocation

Overexpression of Ptch1 significantly inhibits cell growth [131, 132], while in *Ptch1*^-/-^ cells, there is an observed increase in nuclear cyclin B1 and Cyclin D1 levels [132]. Barnes and colleagues demonstrated that Ptch1 interacts with Cyclin B1, sequestering it at the membrane to prevent cell cycle progression. Binding of Hh ligand to Ptch1 disrupts this sequestering, facilitating the translocation of Cyclin B1 to the nucleus [133, 134]. This observation is consistent with developmental findings, where downregulation of Shh induces the redistribution of Cyclin B1 to the apical surface in colocalization with Ptch1 [135], and it has been shown in vivo that Ptch1 regulates the subcellular localization of Cyclin B1 in epithelial cells [127]. Several Ptch1 mutants associated with BCC (Q688X [134], C727VfsX736, C727VfsX745, and S733IfsX736 [136]) exhibit loss of binding to Cyclin B1, and the overexpression of these mutants leads to increased cell cycle progression and Hh pathway activation independent of Shh stimulation. Furthermore, GRK2, while primarily known for its role in Smo phosphorylation, associates with Ptch1 and reduces its interaction with both Ptch1 and Cyclin B1, thereby attenuates the inhibitory effects of Ptch1 on cell cycle progression [137].

### PKA phosphorylates several centrosomal proteins for cell cycle progression

PKA regulates a wide array of cellular responses, and dysregulation of PKA signaling has been implicated in various human diseases, with recent studies identifying mutations in PKA associated with several cancers [138–143]. PKA is tethered to specific subcellular compartments through interaction with A kinase-anchoring proteins (AKAPs), which compartmentalize cAMP as well, resulting in selective activation of PKA. Within the context of the Hh pathway, G-protein-coupled receptor 161 (Gpr161) serves as an AKAP for type I PKA regulatory subunits and is also a target of PKA phosphorylation [144, 145]. This phosphorylation event facilitates the removal of Gpr161 from cilium, thereby fine-tuning PKA activity [146, 147].

PKA is localized at the centrosome in various cell types [148–150], where it is tethered by proteins such as AKAP350 [151], AKAP450 [152], centrosome and Golgi localized PKN-associated protein (CG-NAP) [153], and pericentrin [154]. The delocalization of PKA from the centrosome phenocopies the effects of ciliary ablation and adenylate cyclase 3 (AC3) knockdown, indicating that centrosomal PKA functions as a downstream effector of ciliary-produced cAMP [155]. The centrosome serves as a platform for cAMP/PKA signaling downstream of the primary cilium [156] and plays a pivotal role in processes such as DNA replication, centrosome duplication, cell division, and cytoskeleton rearrangements [157]. PKA phosphorylates centrin to regulate centriole separation and centrosome duplication [158], interacts with pericentrin [154], and phosphorylates cytosolic dynein [159], both of which are critical for microtubule nucleation [160]. Furthermore, PKA is found in complexes with cell cycle-dependent kinase (CDK)5 regulatory subunit-associated protein 2 (CDK5RAP2), AKAP9, and end-binding 1 (EB1), where AKAP-associated PKA activity is essential for stabilizing cortical microtubules [161]. PKA also phosphorylates nuclear distribution protein nudE homolog 1 (Nde1) and nuclear distribution protein nudE-like 1 (Ndel1), facilitating the release of lissencephaly 1 (Lis1) and dynein from the centrosomal localized Lis1-dynein-Nde1-Ndel1 complex, thereby coupling the nucleus and centrosome [162–164].

In addition to its roles in the Hh pathway through the phosphorylation of Gli proteins [92, 165] and its participation in the Ptch1-ArhGAP36-PKA-inversin-Smo axis in the primary cilium or at the ciliary base [89], PKA also binds to the Gpr161 AKAP motif with regulatory subunits and the protein kinase inhibitor motif of Smo with its catalytic subunit [146, 166, 167]. Furthermore, PKA localizes to the centrosome, where it regulates centrosome duplication and nucleus-centrosome coupling by phosphorylating centrosomal proteins and Sufu [168]. Consequently, PKA is considered a “star” protein for investigating the crosstalk between the Hh pathway and cell cycle progression. Further research is warranted to elucidate the reasons behind the existence of different regulatory modes related to the Hh pathway, both within and outside the primary cilium, and to explore the cooperative roles of PKA in modulating both the Hh pathway and cell cycle dynamics.

### Sufu suppresses the G1-S transition

In ciliary-deficient *Kif3* or *intraflagellar transport* (*IFT*)*88* depletion mutants, Sufu retains its repressive effects by maintaining reduced levels of Gli, while inactivation of Sufu leads to increased activity of Hh pathway [95, 169]. Furthermore, addition of *Drosophila* Sufu recovers Gli protein function in *Sufu* depleted mammalian cells [169]. Thus, the primary cilium is not essential for Sufu-mediated inhibition of the Hh pathway.

Despite regulating Gli distribution and transcriptional activity, our recent results show that Sufu also facilitates the phosphorylation of centrosomal protein 110 (CP110) at the centrosome by CDK2 and promotes the degradation of Cdt1 within the nucleus [168]. Acting as a negative switch for both initiations of centrosome duplication and DNA replication, Sufu levels must be tightly controlled and coordinated with the cell cycle to permit only a single round of centrosome duplication and DNA replication. The accumulation of the Sufu-Gli complex in the nucleus is associated with its localization at the ciliary tip [170], occurring just before the upregulation of nuclear Cdt1. We propose that Sufu releases its repression by translocating from the mother centriole to the ciliary tip and from the nucleus to the cytoplasm simultaneously or by directly targeting nuclear Sufu for proteolysis, thereby enhancing the coupling between the initiation of centrosome duplication and DNA replication. Taken together, further research is required to elucidate the mechanisms by which Hh pathway components including Sufu, contribute to the regulation of cell proliferation and cell cycle progression.

### Functions of Hedgehog-containing extracellular vesicles in development and disease

The Hh ligand is released via the budding of vesicles from the plasma membrane [171]. Hh-containing extracellular vesicles (EVs) derived from peripheral blood have been shown to enhance vasculogenesis and nitric oxide (NO) production capacity of endothelial progenitor cells [172]. Furthermore, these EVs stimulate the expression of messenger RNA and proangiogenic factors, such as vascular endothelial growth factor (VEGF), thereby promoting angiogenesis [173]. Hh ligand has also been detected in liver-derived EVs, where it plays a role in vascular remodeling during cirrhosis[174]. Additionally, Hh has been observed to originate from apical microvilli budding on the surface of the ventral node, where it regulates left-right determination during development [175]. Notably, platelets contain epithelial Hh in the form of EVs, which are subsequently delivered to the perinatal dentate gyrus (DG) [176]. Therefore, Hh-containing EVs exhibit bioactive functions that regulate downstream signaling pathways.

Other components of the Hh pathway, such as Ptch1 and Smo, have been identified on EVs released by cervical cancer cells (CaCx) [177]. The localization of Ptch1 at the contact site between presenting and receiving cytonemes necessitates an EV fusion mechanism, with the transport of Ptch1 relying on the formation of MVBs via the ESCRT machinery [178]. Additionally, Gpr161 is released in retrieval mutants at the ciliary tip, regulated by an actin network [179].

Recent advancements in nanoflow cytometry and atomic force microscopy have facilitated the quantification and mapping of subpopulations of EVs [180, 181]. The label-free identification of EVs using core-shell nanoparticles has the potential for high-throughput identification, significantly reducing acquisition time [182]. Fluorescence correlation spectroscopy enables imaging at the single-particle level [183]. These technical innovations in characterizing molecular signatures strongly support ongoing research into the cellular origin and functional roles of EVs.

### Hh pathway in cancer

Cancer remains one of the leading causes of mortality globally. Uncontrolled cell proliferation and deregulation of the cell cycle are hallmark characteristics of cancer cells and neoplastic development. In recent decades, the aberrant activation of the Hh pathway has been implicated in critical processes such as carcinogenesis, cancer progression, metastasis, and tumor pathogenesis [184–192]. This dysregulation has been associated with various cancers, including those affecting the brain, craniofacial complex, thyroid, lung, breast, stomach, liver, pancreas, kidney, colon, muscle, hematopoietic system, and skin (Table 1). These findings underscore the importance of the Hh pathway in the etiology of a diverse array of malignancies.

**Table 1:** Cancers caused by dysregulation of the Hh pathway.

Cancer position	Cancer type	Molecules related in Hh pathway
Brain	Glioma [351]	Gli2 (↓), Shh (↑)
	Medulloblastoma [209, 210]	Ptch1 (↑), Shh (↓)
Craniofacial complex	Odontogenic karatocytes [352]	Ptch1 (↑), Smo (↑)
	Oral squamous cell carcinoma [353]	Gli1 (↑), Ptch1 (↑), Shh (↑), Smo (↑)
Thyroid	Thyroid cancers [354]	Gli2(↑), Ptch1 (↑), Shh (↑), Smo (↑)
Lung	Lung cancer [256, 355, 356]	Gli2 (↑), Shh (↑)
Stomach	Gastric cancer [357, 358]	Gli2 (↑), Shh (↑)
Liver	Cholangiocarcinoma [359, 360]	Gli1 (↑), Gli2 (↑)
	Hepatocellular carcinoma [192, 361, 362]	Gli1(↑), Gli2(↑), Ptch1 (↑), Smo (↑)
Pancreas	Pancreatic cancer [185, 363]	Gli1(↑), Shh (↑), Smo (↑)
Kidney	Renal cell carcinoma [364–366]	Gli1 (↑), Gli2 (↑), Shh (↑), Smo (↑)
Colon	Colorectal cancer [367]	Ihh (↓), Shh (↑),
Muscle	Rhabdomyosarcoma [368]	Gli1(↑), Ptch1 (↑)
Hematology	Hematological malignancy [369, 370]	Dhh (↑), Gli1 (↑), Smo (↑)
Skin	Basel cell carcinoma [198, 199]	Gli1 (↑), Ptch1 (↑), Shh (↑)

Tumors are comprised of cancer cells alongside surrounding stromal components, including endothelial cells, fibroblasts, microvascular elements, and other noncellular constituents. These components play a critical role in tumor initiation, progression, and resistance to anticancer therapies [193]. Ligand-dependent Hh pathway activation has been implicated in a diverse array of cancers, many of which do not exhibit mutations in Hh pathway components and can be effectively targeted by Hh inhibitors. Hh ligands produced by cancer cells and/or their stromal environment contribute to the maintenance of an undifferentiated proliferative state. Consequently, the activity of the Hh pathway can influence tumor growth through both cell-autonomous mechanisms (acting directly on cancer cells) and non-autonomous mechanisms (acting on stromal components), highlighting its multifaceted role in cancer biology.

Mutations in Hh pathway components, including Ptch1, Smo, and Sufu, lead to ligand-independent activation of the pathway. BCC is the most prevalent skin cancer in the Western world, with somatic mutations in Ptch1 and Smo frequently identified in sporadic cases [105, 194–199]. Another cancer type characterized by Hh pathway mutations is MB, where mutations in nearly all canonical Hh components, including Ptch1, Smo, and Sufu, are commonly observed [118, 189, 200–210]. Besides BCC and MB, Hh mutations have also been detected in several other cancers, albeit at lower frequencies. (Table 1). These cancers often depend on ligand-dependent signaling due to elevated Hh ligand expression.

Abnormal activation of the Hh pathway contributes not only to the unchecked growth of cancer cells but also to the establishment of an immunosuppressive tumor microenvironment [193]. In contrast, the Hh pathway and Gli take a tumor-suppressive function in Neuroblastoma, which is the most frequent solid tumor in infants and young children [211]. The mechanisms underlying Hh pathway activation in cancer are multifaceted, and both ligand-dependent and -independent pathways may collaboratively promote cancer progression [212, 213]. Consequently, further investigations are essential to elucidate the precise mechanisms of the Hh pathway in cancer, which may lead to the identification of improved therapeutic targets.

## Primary cilium is required for response to the Hedgehog pathway

The Hh pathway is transduced and regulated within the primary cilium, a microtubule-rich protrusion on the apical surface of nearly all vertebrate cell types [214]. In most mammalian cells, the primary cilium typically measures approximately 150–350 nm in diameter and ranges from 1 to 10 μm in length [215]. Core components of vertebrate Hh signaling, including Ptch1, Smo, Sufu, and Gli family, dynamically localize to the primary cilium [216]. Mice that exhibit defects or complete absence of primary cilium demonstrate loss-of-function or altered ratios of Gli activators to repressors, underscoring the critical role of the primary cilium in the proper functioning of the Hh pathway [217].

### Ciliary structure

Eukaryotic cilia are evolutionarily conserved hair-like organelles that project from the apical surface of nearly all cell types [218]. The core structure of the cilium, known as the axoneme, features a 9 + 2 (0) microtubule architecture: two singlet microtubules (only exist in motile cilium) and nine surrounding doublet microtubules [219] extending from the basal body, which is transformed from the mother centriole that contains triplet microtubules along with subdistal and distal appendages [220]. This review focuses specifically on the primary cilium, which functions in transducing the Hh pathway. For information on the features and functions of motile cilia, we direct readers to recent review articles [221–223].

The microtubules of the axoneme serve as tracks for intraflagellar transport (IFT), a specialized transport system [224, 225] essential for the assembly and disassembly of the primary cilium, thereby regulating ciliary dynamics [226, 227]. During IFT, large cargo proteins and motor complexes associate with linear arrays known as IFT trains, which move in both anterograde and retrograde directions along the axoneme. This review will not address the composition or imaging of the IFT system; interested readers are encouraged to consult recent literature on these topics [224–226, 228–230].

Future research should investigate how Hh pathway-related cargo is loaded onto or discharged from IFT trains, the mechanisms of motor attachment or activation, and the regulatory processes that ensure effective signaling transduction. A multidisciplinary approach to studying IFT could provide insights into these critical questions.

### Compartments in primary cilium

**Fig. 2:**
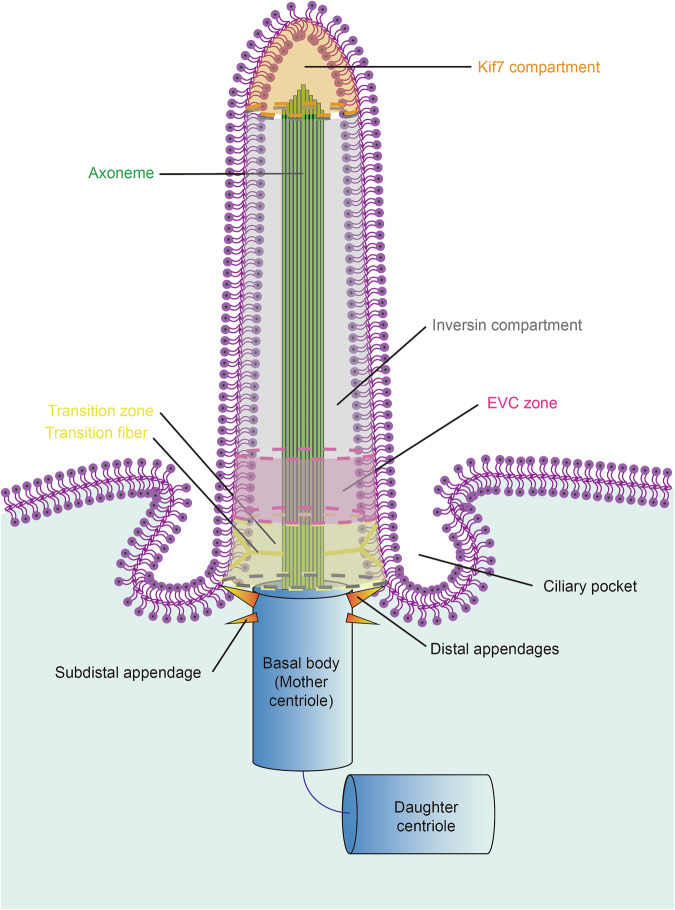
Overview of Ciliary compartments. The primary cilium is a microtubule-based structure consisting of a basal body (converted from the mother centriole), an axoneme that is comprised of nine triplet microtubules, and several compartments within the ciliary membrane. The basal body anchors to the apical surface of a cell with the help of the distal appendages. At the ciliary base, there is a ciliary pocket, which is the ciliary-oriented vesicle docking site. The transition zone and Y-shaped transition fibers (yellow) are located right above the mother centriole and act as a “ciliary gate” that controls proteins translocating in and out of the primary cilium. Above the transition zone is the Ellis-van Creveld syndrome (EVC) zone (magenta), where Smoothened (Smo) is sufficient to activate the downstream pathway in complex with both EVC and EVC2. The inversin compartment (gray), in which protein kinase A (PKA) phosphorylated inversin binds with Smo, starts from the ciliary base to the subdistal ciliary tip. In the ciliary tip Kif7 compartment (orange), the Suppressor of fused (Sufu)-glioma-associated oncogene (Gli) complex dissociates. The primary cilium conducts a set of ciliary compartments to complete its functions more specifically.

The primary cilium serves as a specialized signaling transduction center [231], possessing a composition distinct from the rest of the cell [232]. Despite this distinctiveness, the ciliary membrane is continuous with the plasma membrane, and the ciliary axoneme consists of a proximal segment and a distal segment, with tubulin that may or may not be polyglutamylated [233]. To facilitate compartmentalization and maintain this unique composition, the proximal region of the primary cilium features a transition zone that acts as a ciliary gate, regulating the movement of proteins in and out of the cilium [234, 235]. The transition zone is characterized by Y-shaped transition fibers that connect the ciliary membrane to the axoneme, thereby organizing a diffusion barrier [235, 236]. Additionally, the distal appendages of the basal body may prevent inappropriate vesicle entry into the primary cilium. Precise localization of proteins within the primary cilium is crucial for effective signal transduction (Fig. 2).

Precise localization of proteins within the primary cilium is critical for effective signal transduction. The “Ellis-van Creveld (EVC) zone” located at the base of the primary cilium (basal body), defines a compartment essential for the binding of Smo to EVC and EVC2 (Fig. 2). This interaction is required for the activation of Gli2, the recruitment of Gli3 to the ciliary tip, and the dissociation of the Sufu-Gli3 complex [237–240].

Additionally, the primary cilium contains the “inversin compartment” (Fig. 2), characterized by the localization of inversin [241, 242]. Defects in inversin are associated with left-right asymmetry, nephronophthisis-2, and age-related macular degeneration (AMD) [243, 244]. Within this compartment, inversin assembles into a fibrillar structure [245, 246].

Kif7, a kinesin-4 family protein, acts as a conserved regulator of the Hh pathway and localizes to the ciliary tip, where it modulates ciliary length and structure [233]. Kif7 interacts with the plus ends of microtubules, reducing the rate of microtubule growth and inducing catastrophe, thereby establishing a ciliary tip compartment (Fig. 2) where the disassociation of the Sufu-Gli complex and their activity can be appropriately regulated [247].

**Fig. 3:**
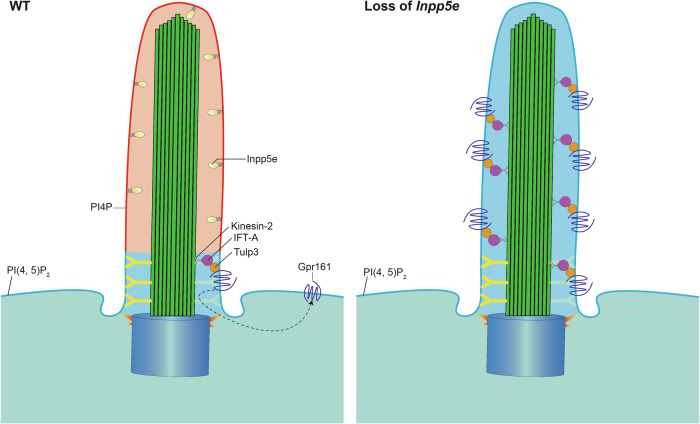
Phosphoinositide compartment. Inpp5e dephosphorylates PI(4, 5)P_2_ to the PI4P in the primary cilium, which induces ciliary exclusion of Gpr161, a negative regulator of the Hh pathway with help of Tulp3, IFT-A, and kinesin-2. In the absence of Inpp5e, ciliary PI(4, 5)P_2_ levels increase, lifting the ciliary Gpr161.

Phosphatidylinositol 4, 5-bisphosphate [PI(4, 5)P_2_] is essential for nearly all membrane processes at the plasma membrane, whereas phosphatidylinositol 4-phosphate [PI(4)P] is predominantly found on the ciliary membrane, excluding the proximal end. In contrast, PI(4, 5)P_2_ is distributed in the proximal primary cilium and the plasma membrane [248, 249]. This differential lipid distribution is regulated by inositol phosphoinositide 5-phosphatase e (Inpp5e), which dephosphorylates ciliary PI(4, 5)P_2_ to generate PI(4)P. Loss of Inpp5e leads to an accumulation of PI(4, 5)P_2_ in the primary cilium, recruiting PI3K, platelet-derived growth factor receptor alpha (PDGFRα) [250, 251], AurA [252], Tubby-like protein 3 (Tulp3) [249], and PI(4, 5)P_2_-binding actin regulators [253], thus accelerating ciliary disassembly and actin polymerization. Additionally, Gpr161 accumulates in primary cilium lacking Inpp5e, resulting in elevated cAMP levels. Hence, Inpp5e establishes a phosphoinositide compartment that limits Hh negative regulators within the primary cilium, permitting ligand-induced Hh pathway transduction (Fig. 3).

Ciliary compartments are crucial for various functions, including protein distribution control, local second messenger supply, membrane identity, and regulation of signaling cascades. Despite the absence of reported cholesterol metabolism enzymes in the cilium, the enrichment of cholesterol in the ciliary membrane compared to the plasma membrane remains enigmatic. Understanding the mechanisms that establish and maintain these ciliary compartments continues to pose challenges in ciliary biology.

## Hh pathway and primary cilium in the cell cycle

### Cell cycle regulation on Hh pathway activity

In most somatic cell lines, our previous findings indicate that the Hh pathway is inactive during the G2 phase due to the phosphorylation and degradation of Gli1, but it reactivates during the G1 and S phases of the subsequent cell cycle, playing a role in regulating the expression of G1-S transition transcription factors, such as Cyclin E. Gli1 expression is downregulated through Polo-like kinase (Plk)1 phosphorylation during the G2 phase, resulting in decreased Sufu-dependent nuclear retention of Gli and subsequent Hh pathway activation [33].

In the Shh-subtype medulloblastoma 55 (SMB55) cell line and the primary cerebellar granule neuron precursors (GNPs), from which SMB55 cells are derived, it has been shown that these cells often maintain cilia beyond the G1 phase into S phase [122]. The Hh pathway activates GNP proliferation through signaling cascades within the primary cilium [122, 126, 254, 255]. Knocking out *Kif3a* to remove the primary cilium inhibits SMB55 cell proliferation and prevents tumorigenesis in various mouse genetic models [255]. SMB55 cells require primary cilium for proliferation and cell cycle determination. SMB55 cells are reliant on the primary cilium for both proliferation and cell cycle determination. Hh pathway activity in the previous cell cycle or during the G1 phase of the current cycle induces G1-S transition and cell cycle entry [122]. Consequently, Cyclin D1 accumulation in the G2 phase is considered indicative of Hh pathway-dependent cell cycle progression, with SMB55 cells and GNPs serving as a paradigmatic system for studying Hh regulation of the cell cycle via the primary cilium.

Further research is needed to elucidate how Hh pathway activity coordinates with cell cycle kinases to regulate the cell cycle and identify potential Hh pathway targets responsible for cell cycle regulation.

### Assembly and disassembly of the cilium during the cell cycle

The primary cilium is a dynamic organelle assembled and disassembled in coordination with the cell cycle and developmental signals [256]. In most somatic cell lines, the primary cilium disassembles before mitotic entry and reassembles at the end of mitosis [257, 258]. The ciliary length is determined by the balance between ciliary assembly and disassembly [218]. Ciliary maintenance is facilitated by IFT, which involves the coordinated movement of anterograde kinesins (IFT-B complex) and retrograde dynein (IFT-A complex) [224–226, 228].

In differentiating granule cell neurons, primary cilium undergoes disassembly through a process termed “deconstruction”, which is distinct from the premitotic ciliary resorption observed in proliferating GNPs [259]. Ott and colleagues categorized this novel type of ciliary disassembly into three classes: pocket cilium, surface cilium, and concealed cilium [259]. This ciliary deconstruction process may serve as a model for the developmental removal of cilia in other tissues, highlighting the dynamic nature of ciliary regulation during cellular differentiation.

Ciliogenesis, the process of building a primary cilium, is an orchestrated event during which polarized membrane vesicles traffic to and subsequently fuse with the plasma membrane [218, 260, 261]. The primary cilium emanates from a specialized centrosomal mother centriole, known as the basal body, which contains specialized structures at its distal end that regulate critical aspects of ciliogenesis and function at the apical surface of most vertebrate cells. Transition fibers, while acting as the boundary between the ciliary and other cellular compartments, mediate the docking of the basal body to the plasma membrane or vesicles during the early stages of ciliogenesis, assisted by the distal appendages. Additionally, the subdistal appendages facilitate the positioning of the primary cilium by anchoring cytoplasmic microtubules [262, 263].

**Fig. 4:**
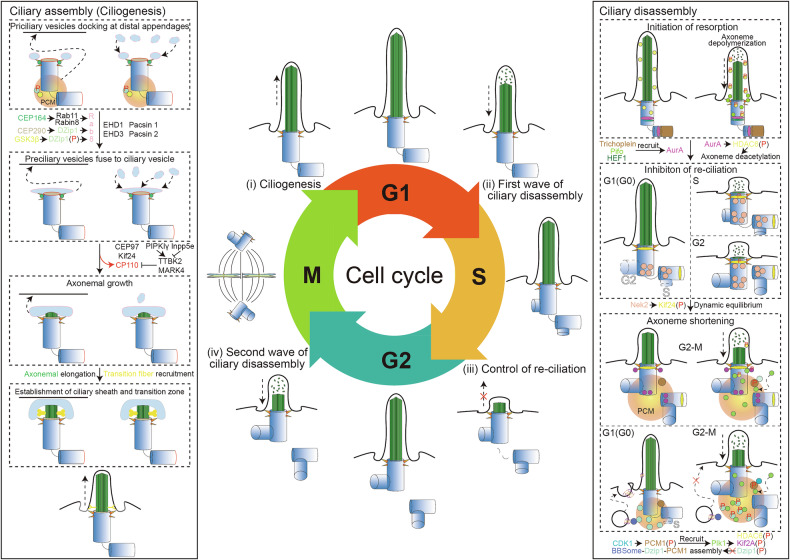
Primary cilium and cell cycle. The ciliary cycle consists of several steps that are linked to the cell cycle. **i** Ciliogenesis. Ciliogenesis is also called ciliary assembly and occurs after cytokinesis in the G1/G0 phase. Centrosomal protein (CEP)164, CEP290, Glycogen synthase kinase 3β (GSK3β), and DAZ-interacting Protein 1 (Dzip1) recruit Rab8-coated vesicles to the distal appendages of the mother centriole to form the ciliary vesicle with help of Eps15 homology domain (EHD)1, EHD3, and their partners Pacsin 1 and Pacsin 2. Subsequently, Tau tubulin kinase 2 (TTBK2) and microtubule affinity-regulating kinase 4 (MARK4) phosphorylates centrosomal protein 110 (CP110) and promotes its release from the distal end of the mother centriole, which lifts inhibition on axonemal elongation. The ciliary sheath and transition zone are then established, and the mother centriole converts to the basal body which anchors to the apical surface of the cell with the distal appendages. **ii** First wave of ciliary disassembly. During the G1-S transition, Trichoplein, Pitchfork (Pifo), and human enhancer of filamentation 1 (HEF1) recruit Aurora A (AurA) to phosphorylate Histone deacetylase 6 (HDAC6), which deacetylates the axoneme of the primary cilium and thus promotes ciliary assembly. **iii** Control of re-ciliation. During both the S and G2 phase, never in mitosis A (NIMA)-related kinase 2 (Nek2) phosphorylates Kif24 and ensures the active state of Kif24, which induces ciliary disassembly for inhibition of re-ciliation. **iv** Second wave of ciliary disassembly. During the G2-M transition, cycle-dependent kinase 1 (CDK1) phosphorylates the pericentriolar material 1 (PCM1), which recruits polo-like kinase 1 (Plk1). Plk1 then phosphorylates HDAC6, Kif2A, and Dzip1 for axoneme deacetylation, elevated microtubule-depolymerizing activity, and both disassembly of the BBSome-Dzip1-PCM1 complex and removal of Dzip1 and the BBSome from the centriolar satellite, respectively. All these processes mediate ciliary disassembly.

Ciliogenesis is a multi-step process, which typically starts at the G1/G0 phase of the cell cycle [264] (Fig. 4). Upon exiting the cell cycle, the first regulatory step of ciliogenesis involves the migration of the mother centriole to the cell surface, during which it forms a basal body to nucleate the primary cilium [260]. The second step occurs as the mother centriole extends the axoneme with the assistance of ciliary vesicles derived from the Golgi apparatus [265]. Time-lapse imaging has revealed this dynamic process [266]. Vesicles are transported to the mother centriole via the sequential action of dynein and myosin-5A [267]. Subsequently, distal appendage vesicles accumulate and fuse to form a membranous cap, with additional vesicles recruited by Eps15 homology domain (EHD)1 and EHD3 [265], along with their partners Pacsin 1 and Pacsin 2 [268], to enlarge this cap for ensheathing the growing axoneme (Fig. 4). The assembly and extension of the axoneme beneath this cap require the IFT system, selective import through the ciliary gate, and membrane trafficking. Rab GTPases, which are regulated by corresponding guanine nucleotide exchange factors (GEFs) and GTPase-activating proteins (GAPs), convert Rab proteins between active (GTP-bound) and inactive (GDP-bound) states [269]. The Rab8–Rab11 GTPase cascade then regulates membrane trafficking through donor membrane vesicularization and subsequent fusion with the acceptor membrane (Fig. 4). The assembly of distal appendages on the mother centriole involves the recruitment of several proteins, including centrosomal protein (CEP)83, CEP89, FBF1, SCLT1, and CEP164 [270]. CEP164 is involved in recruiting Rabin8 to promote the centrosomal activation of Rab8 and is indispensable for the docking of vesicles at the mother centriole [271]. The third step of ciliogenesis is the docking of the basal body to the plasma membrane, facilitated by the distal appendages [270, 272]. Rab8 and ADP-ribosylation factor-like 13B (Arl13B) contribute to ciliary membrane growth and the trafficking of ciliary proteins toward the primary cilium, while Oral-facial-digital syndrome 1 (OFD1) is removed from centriolar satellites through selective autophagy induced by serum starvation for ciliogenesis [273].

Several cell cycle proteins are involved in regulating ciliogenesis [274] (Table 2), including CEP290 [275], Talpid3 (Tpld3) [276], CP110, CEP97 [277] and GSK3β [278]. CP110 has been shown to form a cap above the growing microtubules at the distal end of centrioles [279], and also negatively regulate ciliogenesis upon binding with CEP97 [277], Kif24 [280], Tpld3 [276] and CEP290 [275]. Two kinases have been reported to possess the ability to exclude CP110 from the mother centriole. One is Tau tubulin kinase 2 (TTBK2), which is recruited to the mother centriole by CEP164 [281]. TTBK2 phosphorylates M-phase phosphoprotein 9 (MPP9), which is recruited to the distal end of the mother centriole by Kif24 and inhibits ciliogenesis by recruiting CP110 and CEP97 [282], thereby promoting the removal of CP110 from the mother centriole. The other kinase is microtubule affinity-regulating kinase 4 (MARK4), whose depletion also results in the removal of the CP110-CEP97 inhibitory complex from the mother centriole [283] (Fig. 4). During the metaphase-anaphase transition, a fraction of active GSK3β localizes to the centrosomal area, where it phosphorylates DAZ-interacting Protein 1 (Dzip1) and subsequently activates Rab8 for ciliogenesis following mitosis [284].

**Table 2:** Cell cycle proteins take part in regulating ciliary assembly and disassembly.

Cell cycle proteins	Related cell division processes	Function
Anaphase-promoting complex	key ubiquitin E3 ligase [297, 371–379]	Ciliary disassembly [297]
Aurora A	Centrosome duplication, centrosome separation, spindle assembly, chromosomal alignment, and cytokinesis, and initial activation of CDK1 [380–383]	Ciliary disassembly [257, 291, 292, 296, 384, 385]
CDK1	Nuclear envelope breakdown (NEBD), laminolysis, chromatin condensation, spindle assembly, G2/M transition [386–394]	Ciliary disassembly [258]
Cenp J	Centrosome duplication, centriole elongation [279, 395]	Ciliary disassembly [396]
Centrin2	Centrosome duplication, DNA damage repair, and cytokinesis,	Ciliary assembly [397]
CP110	Centrosome duplication, centriole elongation, centriole assembly [398–401]	Ciliary assembly [276, 277, 280, 282, 402–405]
GSK3β	Spindle dynamics and chromosome alignment [406]	Ciliary assembly [284]
NIMA-related kinases (Nek)1	Centrosome separation, spindle assembly, cytokinesis, [407–409]	Ciliary assembly [408]
Nek2	DNA damage repair, centrosome separation, spindle assembly, cytokinesis [407–412]	Ciliary disassembly [288, 408, 413, 414]
Nek10	DNA damage repair [407–409]	Ciliary assembly [408, 415]
Plk1	Spindle assembly, centrosome maturation, cohesion removal, APC inhibitor inactivation [386, 410–412, 416–418]	Ciliary disassembly [258, 278, 293]
STIL	Centrosome duplication, centriole elongation [395, 419–425]	Ciliary disassembly [426]

Ciliary disassembly has been reported to occur in two waves, with the first wave occurring 1–2 h into the G1 phase following stimulation and the second wave occurring 18–20 h before mitotic entry in retinal pigment epithelial (RPE) [257] and National Institutes of Health (NIH) 3T3 [258] cell line. Ciliary disassembly requires the destabilization and depolymerization of axonemal microtubules. Histone deacetylase 6 (HDAC6) has been identified as a tubulin deacetylase that regulates tubulin stability through reverse acetylation of microtubule [285] and has further been recognized as an effector of serum-stimulated ciliary disassembly [257]. The human kinesin-13 family, which possesses microtubule-depolymerizing activity by inducing a destabilizing conformation of tubulin subunits, consists of Kif2A, Kif2B, Kif2C, and Kif24, and plays a role in the depolymerization of ciliary microtubules [286]. Kif2A is degraded through the ubiquitin-proteasome system during the quiescent G0 phase and localizes to the proximal ends of both centrioles and the subdistal appendages of the mother centriole (Fig. 4), where it promotes the depolymerization of ciliary microtubules and facilitates ciliary disassembly [287]. In contrast to Kif2A, Kif24 acts more as an orchestrated safeguard that prevents aberrant ciliary assembly [288] (Fig. 4). Additionally, several cell cycle proteins participate in the ciliary disassembly process (Table 2).

The calcium-calmodulin active AurA is a well-known centrosomal kinase that regulates mitotic entry [289]. The scaffolding protein human enhancer of filamentation (HEF)1 activates AurA, which subsequently phosphorylates HDAC6 to facilitate the deacetylation of axonemal microtubules [257, 285, 290] (Fig. 4). Two additional AurA activators located at the mother centriole, trichoplein, and Pitchfork (Pifo), also regulate ciliary disassembly [291, 292] (Fig. 4). Furthermore, a Wnt5a- CK1ε-Dishevelled 2 (Dvl2)-Plk1 axis regulates HEF1/AurA-dependent ciliary disassembly [293]. The nuclear distribution element 1 (Nde1), whose level is controlled by CDK5 phosphorylation in G0/G1 cells for subsequent degradation [294], serves to protect trichoplein from ubiquitin-proteolysis [295], thereby enhancing its stabilization [296]. Additionally, AurA binds to and phosphorylates Inpp5e, which promotes the increased 5-phosphatase activity of Inpp5e, resulting in elevated levels of phosphatidylinositol 4-phosphate (PI(4)P) and facilitating ciliary disassembly [252].

Another centrosomal cell cycle kinase, never in mitosis A (NIMA)-related kinase 2 (Nek2), is recruited by pericentriolar material (PCM)1 and is expressed during S and G2 phase to phosphorylate Kif24, ensuring the active state of Kif24, which induces ciliary disassembly and inhibits of re-ciliation [288] (Fig. 4). In addition to promoting centrosome disengagement, the anaphase-promoting complex/cyclosome (APC/C), an E3 ubiquitin ligase, regulates axonemal stability through the modulation of Nek1 activity [297].

Plk1, which is recruited to the pericentriolar matrix by CDK1-phosphorylated PCM1 in a dynein-dynactin complex-dependent manner and phosphorylated by AurA at T210 [298, 299], associates with and activates HDAC6 for ciliary disassembly before mitotic entry [258] (Fig. 4). Plk1 has also been reported to phosphorylate both Kif2A, enhancing its microtubule-depolymerizing activity after serum stimulation [287], and to phosphorylate Dzip1 to facilitate the removal of Dzip1 and the BBSome from the centriolar satellite [278], thereby regulating ciliary disassembly during the G2-M transition (Fig. 4). Thus, the recruitment of Plk1 to the centrosomal area before mitosis significantly initiates the subsequent second wave of ciliary disassembly.

In summary, ciliary resorption during the cell cycle occurs in two waves: the first wave (G1/G0 resorption) is regulated by the AurA/HEF1-HDAC6 cascade, while the second wave (G2-M resorption) is mediated by the PCM1-Plk1-(Kif2A/Dzip1/HDAC6) cascades. Additionally, the Nek2-Kif24 cascade continuously regulates ciliary length during the S and G2 phases. The initial ciliary resorption in the G0/G1 phase is essential for the subsequent G1-S transition, and once cells successfully enter the S phase, the suppression of re-ciliation persists until the second ciliary resorption, which proceeds with the release of the basal body for mitotic spindle formation (Fig. 4). The presence of the primary cilium exhibits an antiproliferative role in certain cancer cases, including both premalignant lesions and invasive cancers [300–302]. Conversely, ciliary-dependent signaling pathways, such as Hh and Wnt, can drive cancer development [203, 303]. Furthermore, ciliogenesis and axoneme stability have been linked to kinase inhibitor resistance in cancer [304], highlighting the importance of a thorough understanding of the ciliary dynamics that coordinate the cell cycle.

### Linking primary cilium to the nucleus

Despite migrating to the cell surface as the template for ciliary axoneme elongation during the G1/G0 phase, the centrosome interacts with the nuclear envelope and nucleic acids to facilitate mitotic spindle formation during mitosis [305]. CEP164 localizes to DNA damage sites and is phosphorylated by both the DNA damage response (DDR)-associated kinases, ataxia telangiectasia-mutated (ATM) and ATM-Rad3-related homolog (ATR), in response to DNA damage. This phosphorylation is associated with the establishment of the G2-M damage checkpoint [306, 307]. In addition to CEP164, proteins localized around the ciliary basal body, including CEP290, which mediates ciliogenesis [308], and Centrin2, which is the core centriolar protein [309], also appears to localize to the nucleus in response to DNA damage. These observations suggest a general mechanism that re-localizes proteins at the ciliary base to the nucleus in response to DNA damage.

P bodies, which are cytoplasmic ribonucleoprotein granules composed of messenger RNA (mRNA), microRNA (miRNA), and proteins in charge of mRNA transport, stabilization, silencing, or degradation, colocalize with the ciliary basal body [310] and proteins involved in ciliogenesis, such as OFD1 and PCM1 [311]. Furthermore, OFD1 is associated with components of the translation machine [312]. Thus, there may be RNA processing and translation occurring at the ciliary base.

Similarities, between the transport mechanisms that translocate proteins from the cytoplasm into either the nucleus or primary cilium, the shape and size of the nuclear pore compared to the ciliary base, and the ciliary localization signal (CLS) compared to nuclear localization signal (NLS) strengthen the linking between the primary cilium and the nucleus. Several proposed CLSs, including the RVxP motif (found in polycystin-2), VxPx motif (found in rhodopsin), and the AxS/AxQ motif (associated with somatostatin receptor 3, serotonin receptor 6, and melanocortin-concentrating hormone receptor 1), have been identified [313, 314]. However, there is no consensus sequence for ciliary localization in ciliary receptors, which contracts with the NLS [229]. Kif17, a member of kinesin-2 that drives the biogenesis, maintenance, and function of the primary cilium through IFT, binds to the nuclear import protein importin-β2 in a manner dependent on the CLS. This binding is suppressed by the ciliary-cytoplasmic gradient of the small GTPase Ran, particularly in the presence of high levels of ciliary GTP-bound Ran (RanGTP). Nucleoporins (NUPs), which are subunits of the nuclear pore, including NUP37 and NUP85 (outer ring), NUP35 and NUP188 (inner ring), NUP93 (linker), and NUP62 and NUP 98 [central phenylalanine-glycine (FG) repeats], have also been localized to the ciliary base, where they negatively regulate the ciliary entry of Kif17. These NUPs colocalize with ciliogenesis-related protein CEP290, further demonstrating the physical and molecular similarity between the ciliary pore complex and the nuclear pore complex [315–319]. During mitosis, NUP62, NUP188, and NUP358 [also known as Ran-binding protein 2 (RanBP2)] translocate to the centrosome, where they influence spindle formation [320–322]. Additionally, NUP188 colocalizes with nuclear mitotic apparatus (NuMA), a protein that is a substrate of AurA and is responsible for tethering mitotic spindle microtubules to spindle poles [320, 323]. Two other NUPs, Tpr [324] and Aladin [325], facilitate the translocation of AurA to spindle poles during mitosis, and NUP358 mediates the binding of the centrosome to the nuclear envelope by recruiting Bicaudal D2 (BICD2) [326]. The nuclear import protein importin is also responsible for the ciliary transport of Kif17 [327] and Gli family proteins [328].

Taken together, during cell cycle progression, the primary cilium and the nucleus share components and mechanisms involved in DDR, RNA processing, and sub-compartmentalization, with the centrosome serving as a structural model at the ciliary base that contributes to ciliary diffusion control during quiescence and is associated with components of the nuclear pole transport machinery. While the numerous similarities between the ciliary gate and the nuclear pore complex, as well as potential reciprocal interactions between these two cellular structures, remain unexplored, further investigation is critical to elucidate their roles in cell cycle regulation.

### Ciliary scission and cell cycle progression

Since there are no protein synthesis or degradation organelles present in the primary cilium, all ciliary proteins are transported in and out of this organelle. In addition to the retrograde transport mechanism of IFT, another pathway for the removal of ciliary components is the release of ciliary vesicles, a process that involves the shedding of vesicles at the ciliary tip and occurs during the G1-S transition.

The conventional retrieval pathway for activated GPCRs from the primary cilium back into the cytoplasm relies on the β-arrestin 2 and the BBSome, a complex of Bardet-Biedl Syndrome (BBS) proteins that forms a membranous coat in association with the Arf-like GTPase Arl6 [329–331]. However, ciliary proteins such as Gpr161 or somatostatin receptor 3 (SSTR3), which have been observed in retrograde IFT, may also undergo ciliary vesicle release due to deficiencies in the retrieval machinery (BBSome) or loss of retrieval motifs [332]. Furthermore, the velocity and capacity of ciliary vesicle release (resorption of the primary cilium in 6 h) [333] demonstrate a significant advantage compared to direct retrieval transport via IFT, which operates at less than 10 molecules per minute [332]. The elimination of dynein-2 has shown only mild phenotypes in *Tetrahymena* [334], suggesting that another mechanism could largely assume the role of removing components from the primary cilium. Together with the prerequisite role of rapid deciliation in the loss of the cilium, as evidenced by live-cell imaging [335], and the limited number of proteins identified to be transported out of the primary cilium through the IFT system, ciliary vesicle release serves as the primary mechanism for cell cycle-dependent ciliary resorption [336, 337].

The function of ciliary vesicle release may be analogous to the disposal of excess material in reticulocytes [338] and axons [339], which do not possess lysosomes. However, the best-studied extracellular vesicles, exosomes, have been shown to carry a set of signaling molecules, including receptors, enzymes, second messengers, and RNAs, and to facilitate communication between cells [340]. Vesicles released from the primary cilium also exhibit similar bioactivity [341] and have been demonstrated to carry proteolytic enzymes that degrade extracellular matrix-like cell walls, aiding in the liberation of daughter cells following mitosis [342]. Like other forms of “reverse topology” membrane budding and scission [343, 344], ciliary vesicle release involves ESCRT [337, 345]. However, unlike the typical mechanisms, ciliary vesicle release utilizes actin polymerization, including F-actin, sorting nexin 9 (SNX9), myosin 6, drebrin, cofilin, and fascin. Actin polymerization is promoted by an increased level of ciliary PI(4, 5)P_2_, which is regulated by the level of ciliary Inpp5e [179, 346]. Growth stimulation and loss of Inpp5e promote ciliary vesicle release at the ciliary tip, whereas AurA inhibition using alisertib suppresses growth-stimulated ciliary scission, and HDAC6 inhibition using tubacin shows only a mild effect [346]. Furthermore, AurA inhibition counteracts growth-stimulated Inpp5e depletion. Thus, the regulation of ciliary vesicle release by AurA and Inpp5e plays a significant role in both ciliary vesicle release and ciliary disassembly during the G1-S transition.

While centrosomes act as a center for intracellular signaling [347, 348], the primary cilium serves as a hub for extracellular signals [256, 349, 350]. More studies are needed to elucidate how ciliary-derived vesicles transduce signaling pathways and what other signals, proteins, miRNAs, and even viruses can be encapsulated.
